# Childhood predictors of cluster A personality disorder traits in adolescence: a seven-wave birth cohort study

**DOI:** 10.1007/s00787-025-02936-x

**Published:** 2025-12-22

**Authors:** Lars Wichstrøm, Hanne Grønli, Jenny Sundbø Walstad, Andrea Raballo, Elfrida Hartveit Kvarstein, Silje Steinsbekk

**Affiliations:** 1 Department of Psychology, The Norwegian University of Science and Technology, Trondheim, Norway; 2 The Department of Mental Health—Acute, Elderly, Child and Adolescent Services, Department of Child and Adolescent Psychiatry, Trondheim, Norway; 3 Department of Psychiatry, University of Lugano, Lugano, Switzerland; 4 Section for Treatment Research, Department for Research and Innovation, Division of Mental Health and Addiction, Oslo University Hospital, Oslo, Norway; 5 Institute for Clinical Medicine, University of Oslo, Oslo, Norway

**Keywords:** Adolescence, Community, Childhood, Cluster A, Prospective

## Abstract

Cluster A personality disorders are hypothesized to have their origins in childhood, but little prospective research exists to support this contention. We investigated whether factors intrinsic to the child, social-relational and environmental factors, and symptoms of other psychopathologies in childhood predict paranoid, schizoid, and schizotypal personality disorder traits at age 16. A sample from two birth cohorts in Trondheim, Norway (*n* = 1,077; 50.9% female) was examined biennially from age 4–16. Cluster A personality disorder traits were assessed with the Structured Clinical Interview for DSM-5 Personality Disorders and regressed on the intercept and growth in child risk and protective factors up to age 14. The prevalence of any Cluster A PD at age 16 was 2.41% (95% CI: 1.12, 3.69); paranoid 1.36% (CI: 0.42, 2.31); schizoid 0.56% (CI: -0.11, 1.23); schizotypal 1.05% (CI: 0.23, 1.87). Elevated and rising levels of odd or eccentric behavior, heightened and increasing neuroticism, low conscientiousness, declining self-esteem, and growing emotional and behavioral difficulties predicted both paranoid and schizotypal features, whereas low and rising levels of insecure attachment predicted paranoid traits only. Schizotypal traits also shared early risk factors with schizoid traits: Problems with emotion regulation and cluster A traits in parents. Several assumed predictors were unpredictive: Having an imaginary friend, disorganized attachment, negative life-events, and autism spectrum symptoms. In conclusion, cluster A traits at age 16 can be predicted by a range of factors already evident in childhood and early adolescence, most notably oddity, symptoms of emotional and behavioral disorders, low self-esteem, social withdrawal, and personality traits.

**Supplementary information:**

The online version contains supplementary material available at 10.1007/s00787-025-02936-x.

## Introduction

Paranoid, schizoid, and schizotypal personality disorders (PDs) comprise Cluster A PDs [1], with an estimated worldwide prevalence of 3.8% [46]. Clinically, individuals with cluster A PDs often appear “odd” or “eccentric” and social avoidance is typically pronounced. The adverse outcomes are numerous, including lack of an intimate partner, failure to live independently, poor academic achievement, unemployment, comorbid psychiatric disorders, and increased suicidality risk [21]. About one-third of those diagnosed with schizotypal disorder have been found to convert to schizophrenia within 20 years [22], and paranoid PD may also increase the risk of later delusional disorder [5]. Even though PDs are typically not diagnosed before adulthood, there is much to indicate that cluster A traits are present already in childhood and adolescence [36], and there is a continuity of cluster A traits from adolescence to adulthood [25]. Despite a widespread belief that Cluster A PDs start in childhood and adolescence [1], most research has relied on cross-sectional or retrospective reports from adults—often patients—and there is limited prospective research to substantiate this claim. As a remedy, the present work aims to test common assumptions about the childhood precursors of paranoid, schizoid, and schizotypal traits at age 16 using biennially collected data from ages 4 to 14 in a birth cohort sample. Even though the heritability of Cluster A disorders and traits is high, with estimates varying between 21% to 72% [27, 28], a substantial proportion of the variation is due to non-genetic factors. Environmental and developmental characteristics may account for part of this variance, and delineating these factors is important for advancing preventive and treatment efforts. A broad range of risks and early manifestations have been suggested, which we address here, spanning (i) those intrinsic to the child, (ii) social-relational and environmental risks, and (iii) other psychopathologies.

### Child factors

Even though engaging in fantasy and magical thinking is normative in childhood, a subset of children may exhibit a somewhat nebulous differentiation between reality and fantasy, causing them to appear odd. One longitudinal study indeed found that high initial levels and increasing oddity from early school age to adolescence predicted adolescent schizotypal traits [14]. Whether such predictions also exist for paranoid and schizoid traits remains undetermined. Relatedly, some children have an imaginary companion, possibly as a result of high fantasy proneness, which is associated with schizotypal features [31], and adults with schizotypal PD more often report having had an imaginary companion in childhood [13]. Cross-sectional [40] and longitudinal [38] studies in adults have found executive functional deficits associated with Cluster A traits, but prospective studies in childhood are lacking. Paranoid ideation, suspiciousness, and bizarre ideas are cross-sectionally associated with low self-esteem, and intensive experience sampling studies report declining self-esteem to predict paranoia in adults [43], suggesting that poor self-esteem in childhood may increase Cluster A PD traits later in life. Moreover, there is ample evidence of continuity between Big Five personality traits and Cluster A traits [39], but whether childhood personality traits predict later Cluster A traits remains unexamined. Finally, findings suggest that difficulties in regulating emotions are associated with schizotypy [19] as well as paranoid thinking [3], but the temporal order of effects from childhood has not been investigated.

### Social-relational and environmental factors

Given that social withdrawal is common to cluster A PDs and that adults with schizotypal PD evidence social skills deficits [44], it is possible that reduced social skills and social withdrawal in childhood forecast cluster A traits in adolescence. Insecure attachment is more prevalent in adults high on paranoid and schizotypal traits [34]. As insecure and disorganized attachment in childhood forecasts a range of psychopathologies [30] and have thus been proposed as a transdiagnostic risk factor, we expect —and are the first to test—whether this extends to Cluster A traits as well. Indeed, due to heritability and potential parenting effects [45], not only enhanced Cluster A, but also Cluster B and C traits in parents should be expected to predict Cluster A traits in adolescence. There is ample evidence that childhood adverse events predict adult Cluster A traits and disorders, also when prospective designs are employed [6, 10]. Whether this prediction extends to a broader set of negative life events remains uncharted, but research is called for [10]. In particular, attention has been drawn to the possibility that victimization from bullying may increase the risk of developing paranoid and schizoid traits, which are correlated in childhood and adolescence [17].

### Psychopathologies

Cluster A PDs co-occur with a range of other psychopathologies, and the reasons for this comorbidity may be multiple, including the possibility that other psychopathologies predispose adolescents to develop Cluster A traits. Indeed, results from the US-based Children in the Community Study showed that childhood depression, anxiety, and disruptive disorders predicted paranoid and schizotypal PDs in adulthood [8], and a small-scale US study also indicated that externalizing problems in middle childhood forecasted paranoid PD traits in adolescence [35]. We investigate whether these findings also extend to schizoid traits. Moreover, autism spectrum disorder (ASD) covaries with Cluster A traits in adults [37] and adolescents [2]. The reasons might include overlapping diagnostic criteria as well as common genetics [29]. Regardless of the reasons for the comorbidity, elevated symptoms of ASD in childhood might predict later Cluster A traits.

### Current study

In conclusion, as shown above, our knowledge of the childhood predictors of cluster A traits remains limited. Crucially, the stability of assumed predictors is only modest to moderate throughout childhood, even among those typically assumed to evince continuity, such as attachment styles [26]. Thus, many children with a high early risk of Cluster A traits will experience a more positive developmental course, whereas other children’s risk may increase further. Therefore, as suggested by others [14], not only the *level* of risk at a specific age but also the *development* of risk throughout childhood will be considered. We will aim to provide novel insights by drawing on data from two birth cohorts studied biennially from age 4 to 16 years, hypothesizing that elevated as well as increasing levels of factors intrinsic to the child (i.e., executive functioning problems, oddity, neuroticism, low extraversion, conscientiousness, openness and agreeableness, poor self-esteem and emotion regulation, and having an imaginary friend), social-relational and environmental factors (i.e., low social competence, social withdrawal, insecure and disorganized attachment, negative life-events, victimization from bullying, and personality disorder traits in parents), psychopathological factors (symptoms of emotional, behavioral, and autism spectrum disorders) will predict higher levels of cluster A traits at age 16.

## Methods

### Procedure and participants

As part of the Trondheim Early Secure Study (TESS), children born in 2003–2004 in Trondheim, Norway (*N* = 3,456), and their caregivers were invited to participate through a letter enclosed with a routine health screening notice for 4-year-olds. The letter also included the Strengths and Difficulties Questionnaire (SDQ) [18], designed to assess emotional and behavioral difficulties. Of the 3,358 children who attended the clinic, 2,477 families agreed to take part in the study. A total of 176 families were excluded due to language barriers, and 166 were not invited due to procedural errors. To ensure sufficient variation in symptom severity and optimize statistical power, a stratified sampling method based on SDQ scores was used (cut-offs: 0–4, 5–8, 9–11, 12–40), with increasing probabilities of selection across strata (0.37, 0.48, 0.70, and 0.89, respectively). This intentional oversampling was statistically corrected during analysis. Ultimately, 1,250 families were selected for inclusion, and baseline data were obtained from 1,007 children (Mage = 4.7 years, SD = 0.3; 50.9% girls).

**Fig. 1:**
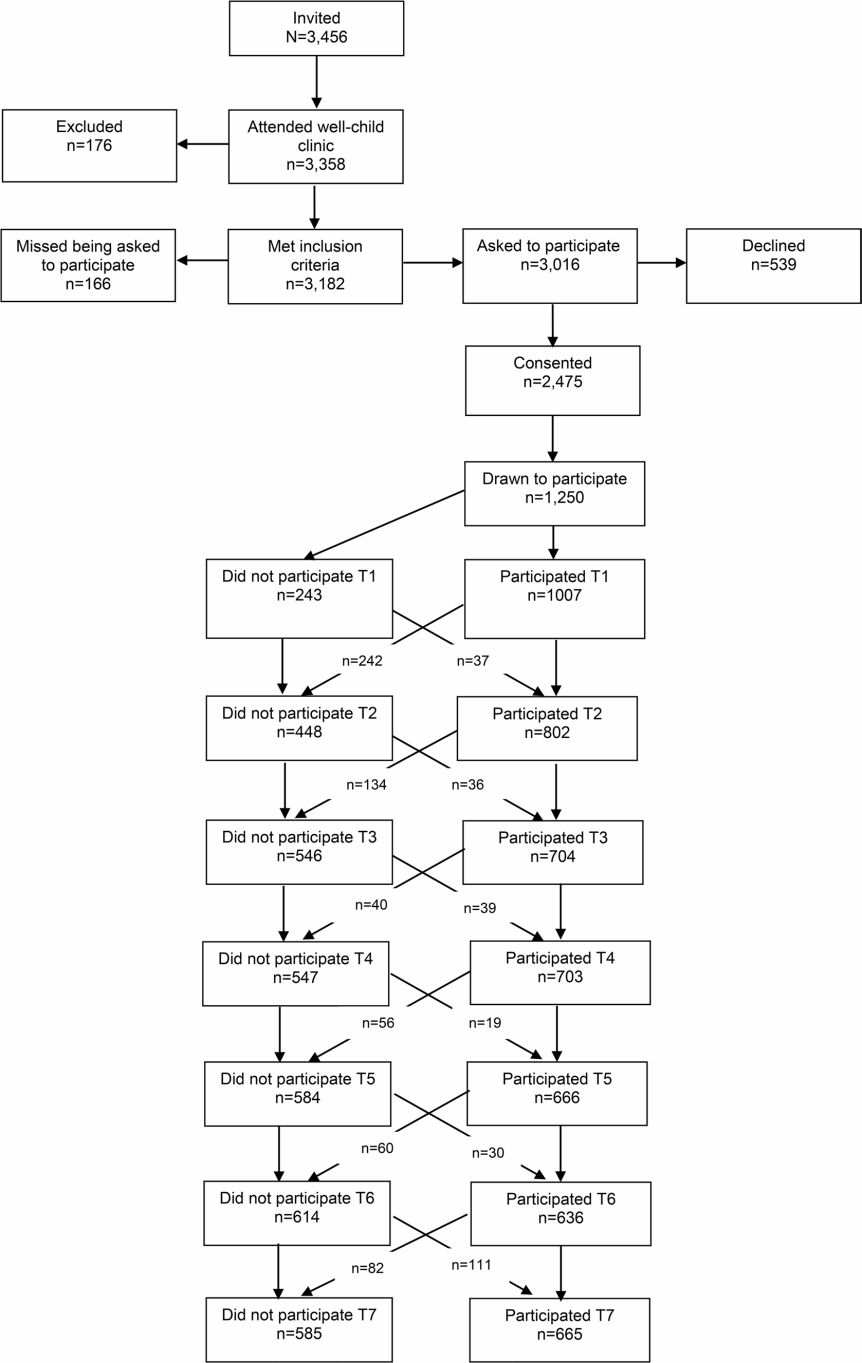
Flow chart of recruitment and follow-up

Participants were followed biennially through age 16 (Fig. 1). The result from Little’s MCAR procedure [29], using all data applied in this work, was consistent with the data being missing completely at random (χ² = 79,081, df = 83,981, *p* = 1.00). The sample was predominantly of Norwegian descent. Most parents were in long-term cohabiting or marital relationships (84.8%), and parental education levels were relatively high (64% of mothers and 52% of fathers held at least a bachelor’s degree; see Supplementary Table 1 for further demographic details). The study protocol was approved by the Regional Committee for Medical and Health Research Ethics in Mid-Norway (approval no. 2019/509). Informed consent was obtained from parents prior to participation, and from adolescents themselves upon reaching 16 years of age, in accordance with Norwegian regulations.

### Measures

#### Cluster A traits

At age 16, Cluster A traits—paranoid, schizoid, and schizotypal—were assessed by interviewing adolescents with the Structured Clinical Interview for DSM-5 Personality Disorders (SCID-5-PD) [16]. Each trait was rated on a 3-point scale: 0 (absent), 1 (subthreshold), and 2 (threshold-level or above), based on both general and specific diagnostic criteria. A composite score was calculated by summing these individual trait scores. In addition, diagnoses were set according to the Diagnostic and Statistical Manual of Mental Disorders, Fifth Edition [1], using traits that scored at the threshold-level or above. Interviewers held at least a bachelor’s degree and had extensive experience with psychiatric assessments of adolescents. To ensure reliability, a subset of 114 interviews was independently rated by trained, blinded coders, resulting in intraclass correlations of 0.90, 0.88, and 0.73 for paranoid, schizoid, and schizotypal traits, respectively.

#### Predictors

An overview of the measured predictors is provided below, with complete psychometric information, instruments, and procedures detailed in the Supplementary Materials.

#### Child level factors

- Oddity: Assessed via seven parent-reported items from the Child Behavior Checklist (CBCL) indicative of unusual perceptions or behaviors (ages 4–14).
- Executive functioning: Evaluated using the Behavior Rating Inventory of Executive Function (BRIEF) (ages 6–14).
- Imaginary companion: Parents reported on imaginary friends at ages 4 and 6 (“No”/”Yes”).
- Self-esteem: Measured using the Self-Description Questionnaire I (ages 6–10) and the Revised Self-Perception Profile for Adolescents (SPPA-R, ages 12–14).
- Personality traits: The Big Five Inventory (BFI) was administered between ages 10–14, covering Conscientiousness, Agreeableness, Neuroticism, Extraversion, and Openness.
- Emotion regulation: Teachers completed the Emotion Regulation Checklist (ERC).
- Social skills: Teacher ratings were collected via the Social Skills Rating System (ages 4–10) and the Social Skills Improvement System (ages 12–14).
- Social withdrawal: Assessed using the Conflicted Shyness subscale of the Child Social Preference Scale, completed by teachers from ages 6 to 14.
- Attachment: Evaluated using the Manchester Child Attachment Story Task (MCAST, ages 4–6) and the Middle Childhood Attachment Strategies (MCAS, ages 10–14).

#### Social-relational and environmental factors

Parental socio-economic position (SEP) was coded on a 6-point scale (1 = unskilled worker, 6 = leader) according to the International Classifications of Occupations [24].

- Serious negative life events: Parents and children (from age 8) reported on a list of 26 stressful or traumatic events (ages 4–14). Events were counted if reported by either source.
- Bullying victimization: Teachers completed the Olweus Bully/Victim Questionnaire (ages 6–14).
- Parental personality disorder traits: Traits were measured via the DSM-IV and ICD-10 personality questionnaire at ages 4, 6, and 14. Cluster-specific sum scores were calculated (A, B, and C; θ = 0.88–0.95.88.95).

#### Symptoms of psychiatric disorders

Parents were interviewed with the Preschool Age Psychiatric Assessment (PAPA) at ages 4 and 6, and both parents and children were interviewed using the Child and Adolescent Psychiatric Assessment (CAPA) from age 8 to 14. Symptom counts were computed for internalizing (e.g., depression, anxiety) and externalizing (e.g., oppositional behavior, conduct problems) disorders. PAPA (but not the CAPA) also included 28 items tapping into the B-criteria for autism spectrum disorder and 4 A-criteria. A score of these items was created at ages 4 and 6.

### Analysis plan

Due to the developmental nature of the predictors under investigation and the expectation of nonlinear changes across time, we employed latent basis growth models [18] in Mplus version 8.11 when linear trajectories provided inadequate fit based on established statistical criteria [23]. This approach permitted the modeling of developmental trajectories that better reflected observed patterns, with parameters fixed at the initial and final time points to guide the shape of change. When instruments needed to be adjusted to accommodate the developmental appropriateness of the measurement (i.e., self-esteem, social competence, and attachment), separate growth curves were estimated for each instrument. Growth parameters were scaled to represent yearly change. We regressed the count of paranoid, schizoid, and schizotypal traits on both latent growth components—the intercept (baseline level) and the slope (rate of change)—which were allowed to covary. Because this implied a large number of tests, we adjusted the p-values according to the false discovery rate [4]. To examine whether the predictors differed in strength across the three PD traits, a Wald test was employed. To account for the sampling design, we applied population weights that reflect the ratio of children in the population to those sampled within each stratum. A robust maximum likelihood estimator (MLR) was employed, which adjusts standard errors for violations of multivariate normality and heteroskedasticity. Missing data were handled according to a full information maximum likelihood (FIML) procedure, leveraging all available data.

## Results

The prevalence of any Cluster A PD at age 16 was 2.41% (95% CI: 1.12, 3.69), with paranoid PD being the most frequent (1.36%, CI: 0.42, 2.31), followed by schizotypal (1.05%, CI: 0.23, 1.87), whereas schizoid disorder was rare (0.56%, CI: −0.11, 1.23). The prevalences of the individual trait scores are listed in Supplementary Table 2. As can be seen, the prevalences of above-threshold scores for schizoid and paranoid PDs were low, whereas the prevalences of below-threshold scores were considerably higher. Paranoid and schizotypal traits were moderately correlated (*r* =.43, *p* <.001), whereas paranoid and schizotypal traits (*r* =.09, *p* =.048) and schizotypal and schizoid traits (*r* =.15, *p* =.052) were weakly associated. The levels and development of the predictors are shown in Supplementary Table 3. Among the child factors, self-esteem increased, while emotion regulation, openness, conscientiousness, and agreeableness all declined. In terms of social-relational factors, social competence increased across childhood and adolescence, whereas the level of secure attachment only increased from age 4 to 6. Disorganized attachment, as well as Cluster B and C traits in parents, decreased. Additionally, symptoms of emotional disorders increased, while experiences of negative life events and bullying victimization declined.

Boys had more symptoms of schizoid traits than girls (B = 0.56, 95% CI: 0.01, 1.11), whereas girls had more schizotypal traits (B = 1.73, 95% CI: 0.91, 2.55). Parental SEP was unrelated to Cluster A traits (paranoid B = −0.40, 95% CI: −0.88, 0.09; schizotypal B = −0.53, CI: −1.08, 0.02; schizoid B = −0.14, CI: −0.41, 0.12), and was therefore not included as a covariate. As can be seen from Table 1, adjusted for sex, paranoid traits were predicted by a range of child factors: high and increasing oddity and neuroticism, low and decreasing self-esteem and conscientiousness during adolescence. In addition, initial high levels of problems with executive functioning and low agreeableness also predicted more paranoid traits. Most factors within the social-relational and environmental domain were unpredictive, except that low social competence in adolescence and initially low and diminishing secure attachment during early childhood (from age 4 to 6) did forecast paranoid traits. An increasing number of symptoms of emotional and behavioral disorders was also predictive.

**Table 1:** Intercept and slope in growth of childhood factors predicting numbers of cluster A personality disorder traits at age 16

		Paranoid			Schizoid			Schizotypal	
Predictors (age range)		Intercept (*p*-value)	Slope (*p*-value)		Intercept (*p*-value)	Slope (*p*-value)		Intercept (*p*-value)	Slope (*p*-value)
Child factors									
Oddity (6–14)		0.17 (0.027)	**0.31** ^c^ **(0.003)**		0.13 (0.011)	0.05 (0.404)		**0.27** ^a ^**(< 0.001**)	**0.32** ^a^ **(0.001)**
Executive functioning problems (6–14)		0.27 (0.010)	0.20 (0.065)		0.13 (0.044)	0.07 (0.445)		**0.33** ^a^ **(< 0.001)**	**0.24** ^a^ **(0.004)**
Imaginary friend (4–6)		− 0.01 (0.762)	− 0.08 (0.102)		− 0.03 (0.295)	− 0.05 (0.367)		0.00 (0.982)	0.01 (0.826)
Self-esteem (6–10)		− 0.01 (0.944)	− 0.22 (0.234)		− 0.17 (0.350)	0.17 (0.472)		0.12 (0.665)	− 0.44 (0.148)
Self-esteem (12–14)		**− 0.24** ^c^ **(< 0.001)**	**− 0.21** ^c^ **(0.001)**		− 0.07 (0.145)	− 0.05 (0.326)		**− 0.16 (0.004**)	**− 0.22** ^a^ **(0.002)**
Openness (10–14)		0.08 (0.149)	0.05 (0.541)		− 0.03 (0.599)	0.02 (0.721)		0.10 (0.042)	0.01 (0.932)
Conscientiousness (10–14)		− 0.17 (0.016)	− 0.13 (0.037)		− 0.07 (0.241)	**− 0.19( 0.002)**		**− 0.32** ^a, b^ **(< 0.001)**	− 0.17 (0.010)
Agreeableness (10–14)		**− 0.29 (< 0.001)**	− 0.12 (0.197)		− 0.12 (0.122)	− 0.03 (0.733)		− 0.15 (0.050)	− 0.04 (0.624)
Extroversion (10–14)		− 0.11 (0.058)	− 0.09 (0.193)		− 0.14 (0.045)	− 0.24 (0.015)		− 0.12 (0.021)	− 0.10 (0.160)
Neuroticism (10–14)		**0.27 (< 0.001)**	0.22 (0.013)		− 0.05 (0.336)	0.10 (0.169)		.**31** ^a ^**(< 0.001)**	0.21 (0.020)
Emotion regulation (6–14)		− 0.10 (0.133)	− 0.23 (0.105)		**− 0.22 (0.001)**	− 0.20 (0.023)		**− 0.20 (0.003)**	− 0.18 (0.076)
Social-relational and environmental factors									
Social competence (4–10)		− 0.15 (0.095)	− 0.03 (0.807)		− 0.07 (0.382)	− 0.09 (0.287)		− 0.16 (0.050)	**− 0.23** ^a, b^ **(0.001)**
Social competence (12–14)		**− 0.22 (0.005)**	− 0.06 (0.498)		**− 0.18 (0.002)**	− 0.07 (0.332)		**− 0.25 (< 0.001)**	− 0.12 (0.081)
Social withdrawal (6–14)		0.02 (0.810)	0.13 (0.164)		0.20 (0.022)	**0.23 (0.006)**		0.14 (0.016)	0.13 (0.119)
Secure attachment (4–6)		−.19^c ^(0.008)	**− 0.23 (0.004)**		0.02 (0.827)	0.05 (0.519)		− 0.08 (0.240)	− 0.09 (0.197)
Secure attachment (10–14)		− 0.12 (0.655)	− 0.01 (0.988)		0.02 (0.946)	0.00 (0.990)		0.19 (0.799)	0.04 (0.988)
Disorganized attachment (4–6)		0.13 (0.231)	0.12 (0.231)		0.09 (0.429)	0.06 (0.511)		0.02 (0.866)	0.04 (0.675)
Disorganized attachment (10–14)		0.05 (0.766)	0.24 (0.160)		0.18 (0.486)	0.32 (0.305)		0.11 (0.315)	0.06 (0.635)
Negative life-events (4–14)		0.25 (0.224)	0.24 (0.278)		0.09 (0.588)	0.06 (0.688)		0.21 (0.180)	0.34 (0.036)
Victimization from bullying (6–14)		0.03 (0.793)	− 0.06 (0.412)		0.12(0.396)	0.15 (0.279)		0.21 (0.015)	0.00 (0.981)
Parental cluster A symptoms (4, 6,14)		0.04 (0.459)	− 0.02 (0.759)		**0.23 (0.001)**	− 0.02 (0.760)		**0.20** ^b^ **(< 0.001)**	0.03 (0.643)
Parental cluster B symptoms (4, 6,14)		0.04 (0.422)	0.07 (0.649)		0.13 (0.038)	0.07 (0.310)		0.12 (0.025)	− 0.02 (0.759)
Parental cluster C symptoms (4, 6,14)		0.03 (0.577)	0.11 (0.335)		**0.18 (0.003)**	− 0.03 (0.8743)		06 (0.279)	0.05 (0.531)
Psychopathology									
Emotional disorder symptoms (4–14)		0.01 (0.907)	**0.37** ^c^ **(< 0.001)**		0.17 (0.044)	0.08 (0.249)		0.02 (0.782)	**0.47** ^a^ **(< 0.001)**
Behavioral disorder symptoms (4–14)		0.15 (0.091)	**0.41** ^c^ **001)**		0.08 (0.210)	0.08 (0.286)		0.19 (0.013)	**0.28** ^a^ **(< 0.001)**
Autism spectrum symptoms (4–6)		0.06 (0.299)	0.11 (0.199)		0.10 (0.110)	− 0.01 (0.859)		0.02 (0.647)	0.10 (0.149)

Note. Standardized estimates. *P*-values ≤ 0.007 are significant at the 5% level, adjusted for the false discovery rate, and are in bold

^a^ schizotypal different from schizoid

^b^ schizotypal different from paranoid

^c^ paranoid different from schizoid, all *p* <.05

Schizoid traits were predicted by more oddity, executive functioning and emotion regulation problems, whereas decreasing conscientiousness, extroversion and emotion regulation also predicted such traits (Table 1). In the social-relational domain, low social competence in adolescence, high and increasing social withdrawal and more Cluster A and C traits in parents proved predictive. In the psychopathological domain, only higher levels of emotional disorder symptoms were slightly predictive.

Schizotypal traits were predicted by various child factors, following a pattern like that of paranoid traits. More specifically, these traits were predicted by high and increasing oddity, executive functioning problems, neuroticism, and low and decreasing conscientiousness. In addition, low and decreasing self-esteem in adolescence was also predictive. In contrast to paranoid traits, schizotypal traits were predicted by several social-relational and environmental factors: high levels of victimization from bullying, increasing social withdrawal and negative life-events, and more cluster A traits in parents. In addition, those with low and decreasing social competence during early to middle childhood (ages 4 to 10) and low levels of social competence during adolescence were also at risk for schizotypal traits. Concerning other psychopathologies, an increasing number of symptoms of emotional disorders were strongly predictive of schizotypal traits, and a high and increasing number of symptoms of behavioral disorders were also predictive.

Tests of difference in predictor strength of the three PDs are noted as superscripts in Table 1. As can be seen, several predictors were more strongly related to paranoid traits than schizoid traits (i.e., oddity, low self-esteem, symptoms of emotional and behavioral disorders). Such a difference also emerged for schizotypal traits (i.e., oddity, executive functioning problems, low conscientiousness, neuroticism, low social competence in childhood, parental cluster A traits, and emotional and behavioral symptoms). However, predictors did not differ for schizotypal and schizoid traits, except that parental cluster A traits were specifically and more strongly related to developing schizotypal traits.

The above results were not corrected for the false discovery rate. Doing so rendered p-values > 0.007 insignificant at the 5% level, which applied to 26 no longer significant predictions (Table 1). Of note, these no longer significant predictions were all in the hypothesized direction.

## Discussion

It is widely assumed that Cluster A personality disorders originate in childhood and adolescence [1]. However, prospective research starting in childhood is lacking to support this claim. We therefore investigated whether the initial level and development of a comprehensive set of child, social-relational, and psychopathological risk factors predicted paranoid, schizoid, and schizotypal traits in adolescence within a birth cohort sample assessed biennially from ages 4 to 16 years. Although results varied somewhat, the predictors of schizotypal and paranoid traits showed substantial similarities. Indeed, only decreasing social competence during childhood and more cluster A traits among parents were more strongly predictive of schizotypal than paranoid traits. Moreover, low secure attachment at age 4 and further decreases towards age 6 predicted paranoid but not schizotypal traits. Several hypothesized childhood risk factors generally predicted both these two traits: low and increasing oddity, executive functioning problems, low self-esteem and social competence in adolescence, low conscientiousness and agreeableness, high neuroticism, and an increasing number of symptoms of emotional and behavioral disorders. Although some similarities existed, many of these predictors were more strongly predictive of schizotypal and paranoid traits than schizoid traits, to which they were often unrelated. The predictors of schizoid traits were fewer; these traits being predicted by decreasing conscientiousness and increasing social withdrawal, low social competence, and more cluster A as well as cluster C traits in parents. Notably, some hypothesized risk factors were not supported, including disorganized attachment, having an imaginary companion, autism spectrum disorder symptoms in childhood, and exposure to serious negative life events.

### Child factors

The finding that higher levels of and increasing oddity predict schizotypal traits replicates one of the few prospective studies on these traits [14]. The fact that also paranoid and schizoid traits were predicted from more oddity also accords with this previous study, which reported childhood oddity to forecast a range of PDs. Growing out of more bizarre and fantasy-driven thinking may be a typical aspect of personality maturation throughout childhood and adolescence. This maturation is also reflected in increasing agreeableness and conscientiousness, as well as decreasing neuroticism [7]. Our results suggest that a disruption in the normative development of personality maturity may make children vulnerable to the development of Cluster A traits.

It is well-known that general cognitive difficulties in childhood predict psychosis spectrum problems in adulthood [32]. Studies on adults suggest that executive function impairments specifically increase vulnerability to the cognitive biases seen in Cluster A disorders. These impairments include deficiencies in impulse control, which interfere with suppressing intrusive paranoid thoughts, as well as shifting difficulties, which hinder the ability to seek and evaluate alternative explanations for a phenomenon [38]. Here, we find support for executive functioning problems already seen in 1 st grade forecasting more traits of all Cluster A disorders in adolescence, widening the support for executive difficulties as a transdiagnostic vulnerability factor.

Studies on adults have implicated poor self-esteem in the development of schizotypy and paranoid thinking [33]. The present study extends this by suggesting that the process may begin in childhood; children whose self-esteem declined over time were at increased risk for paranoid and schizotypal traits. According to a bottom-up perspective on self-esteem, one’s primary source of self-worth is feedback from others. If individuals consistently perceive others as holding negative views of them, even neutral or positive gestures may be questioned and interpreted as hostile—potentially laying the groundwork for paranoid and schizotypal thinking.

Research on adults has associated difficulties in emotion regulation with both paranoid and schizotypal traits [3, 19]. In the present study, we found prospective support for this relationship: problems with emotion regulation in 1 st grade predicted the later emergence of schizoid and schizotypal traits. Although this association was not statistically significant for paranoid traits, the predictive pattern was in the expected direction and did not differ significantly from that observed for schizoid and schizotypal traits. Our measure of emotion regulation (the Emotion Regulation dimension of the ERC) captures not only the presence of positive emotions but also emotional concern for others (e.g., empathy). Thus, it may reflect socio-emotional interest and expressiveness to a greater extent than the primarily intrapersonal processes assessed by adult emotion regulation measures (e.g., cognitive reappraisal). Low ERC scores may therefore indicate early signs of schizoid traits and negative schizotypy.

### Social-relational and environmental factors

Socially competent children tend to have more friendships and deeper social connections, which may provide the necessary feedback to counteract emerging idiosyncratic thoughts and behavior. Social dysfunction, in a broad sense, forecasts early emerging psychotic-like symptoms [41]. Here, we showed that decreasing social competence during the first school years specifically predicted schizotypal traits. Children with schizoid traits have been found to be more solitary than other children [47]. Solitariness may not necessarily imply social dysfunction, but solitary children may lack the feedback from peers that may counteract growing feelings of hostility and social misperceptions [15]. Indeed, socially withdrawn children and those who became increasingly withdrawn through childhood were at increased risk of developing schizoid traits. Of note, it is therefore possible that diminishing social competence and solitariness, in part, reflect early-emerging schizotypal schizoid traits, respectively, that continue into adolescence, and not necessarily that poor social competence or withdrawal is part of the etiology of such traits. Thus, longitudinal studies with measures of PD traits already in childhood could help illuminate the role of social withdrawal in the etiology of schizotypal traits. Although childhood bullying victimization has been retrospectively linked to schizotypy [20] and prospectively to psychotic symptoms [11], this is the first study to demonstrate a prospective link to schizotypal traits already from childhood. Even though there might be selection into becoming victimized by bullying through other risk factors for schizotypal traits, including genetic ones, there is also a likely causal contribution [12], underscoring the need for bullying prevention. Given the heritability of Cluster A traits, a predictive effect of such traits in parents is expected. However, because Cluster A traits are only moderately heritable [28], deficits in parenting associated with parental personality disorders may also contribute to the intergenerational transmission of these traits.

### Childhood psychopathology

The finding that increasing symptoms of emotional and behavioral disorders throughout childhood predicted paranoid and schizotypal traits aligns with the limited existing prospective research [8, 35]. The mechanisms underlying this association remain unclear but may involve shared risk factors for both emotional/behavioral problems and paranoid/schizotypal traits. Additionally, behavioral and emotional difficulties may heighten both interpersonal risks (e.g., low social competence, bullying victimization) and intrapersonal risks (e.g., executive functioning deficits, poor self-esteem), further contributing to the development of these personality traits.

One previous study identified avoidant attachment in childhood as a predictor of later cluster A traits in general [9]. In our data, this link emerged for paranoid traits and not for the other two traits. Moreover, the prediction was stronger for paranoid traits than for schizoid traits, suggesting possible differential pathways within the Cluster A spectrum.

### Unpredictive factors

Several hypothesized predictions were not supported, including disorganized attachment, having an imaginary friend, and symptoms of ASD. For early assessed ASD traits to affect Cluster A traits in adolescence, one would expect continuity in ASD. However, ASD traits [42] exhibit only modest to moderate long-term stability, reducing the potential predictive value. Moreover, our measure of ASD included mainly symptoms from the B criteria (restricted, repetitive patterns of behavior, interests, or activities). A broader coverage of the A criteria (deficits in social communication and social interaction —which align more closely with Cluster A traits—might have improved our ability to detect a predictive effect of early ASD symptoms. Finding no link between negative life events and Cluster A traits, except for increasing negative life-events predicting schizotypal traits, runs counter to other prospective research [6]. Despite our sample exceeding 1,000 participants, the rarity of severe negative life events in the general population may have limited our power to detect associations. Future studies with even larger samples are needed to confirm these findings before drawing firm conclusions.

### Strengths and limitations

This study benefits from several notable strengths, including its use of a population-based birth cohort and repeated biennial assessments that began early in development, allowing for comprehensive tracking of a broad spectrum of potential risk factors. Nonetheless, some limitations warrant consideration. First, our analyses focused on direct associations, only adjusting for sex. Many of the predictors assessed are conceptually related (e.g., extraversion may reflect both social competence and little social withdrawal) and are likely to influence one another dynamically over time. The precise nature of these interrelations, however, remains unclear. Including all variables in a multivariable model could obscure meaningful effects by statistically adjusting for variables that may act as mediators or interact with one another. Future studies utilizing mediation and moderation analyses may help clarify the pathways through which these factors exert their influence and the conditions under which they amplify or buffer the risk of developing Cluster A traits. Second, we assessed Cluster A traits only at age 16. It is possible that these characteristics emerge earlier in development, and the identified predictors may reflect early signs or consequences of emerging Cluster A features. Third, because there is symptom overlap among paranoid, schizoid, and schizotypal personality traits, it is difficult to determine whether the observed predictive associations are due to shared features across these disorders or reflect distinct developmental pathways converging on similar outcomes. Fourth, our measure of ASD was skewed towards restricted, repetitive patterns of behavior, interests, or activities, and to a lesser extent, captured social communication and interaction difficulties. Hence, measures tapping directly into the latter dimension might prove predictive of Cluster A traits. Finally, the sample was predominantly white and from relatively well-educated families, which may limit the generalizability of our findings to more diverse populations or other cultural contexts.

## Conclusions

Cluster A traits are believed to have origins in childhood, but there is a dearth of prospective research starting from this early age to support such a claim. The results from this birth cohort sample, assessed biennially from preschool age to adolescence, revealed that Cluster A traits at age 16 were predicted by a range of child-related factors. Several childhood factors predicted both paranoid and schizotypal traits, most prominently high and increasing oddity, high and increasing neuroticism, low conscientiousness, low and decreasing self-esteem, and increasing symptoms of emotional and behavioral disorders. Schizotypal traits additionally shared predictors with schizoid traits, including deficits in emotion regulation and parental cluster A traits. Some factors were only predictive of specific traits, most notably insecure attachment in early childhood predicting paranoid traits, victimization from bullying forecasting schizoid traits, and parental cluster C traits being associated with later schizoid traits. Genetically informed designs and cross-lagged observational studies may provide further insight into the etiological role of these risks, offering promise for illuminating whether targeting these factors early may reduce the likelihood of developing Cluster A traits later in life.

## Supplementary information

Below is the link to the electronic supplementary material.

Supplementary file 1 (DOCX 54.2 KB)

## Data Availability

No datasets were generated or analysed during the current study.
