# Supplementary material for: Childhood predictors of cluster A personality disorder traits in adolescence: a seven-wave birth cohort study
Source: Eur Child Adolesc Psychiatry. 2025 Dec 22;35(4):1309–19. doi: 10.1007/s00787-025-02936-x (PMC13219160; doi:10.1007/s00787-025-02936-x)
Supplement: Supplementary file 1 — (DOCX 54.2 KB) [file 787_2025_2936_MOESM1_ESM.docx]

**Measures of predictors**

**Oddity. I**n line with prior conceptualizations of early indicators of unusual thinking and perceptual experiences^1^ we created a composite measure of oddity based on parent-reported behaviors using items adapted from the attention and thought problems subscales of the Child Behavior Checklist (CBCL). The measure consisted of seven items, including examples such as “Sees things that aren’t there”, “Strange ideas”, “Daydreams or gets lost in his/her thoughts”. The CBCL was by parents at ages 6-14 and items averaged to make a composite measure oddity (α=.55-.71).

**Executive Functioning**. The teacher most familiar with the child completed the Behavior Rating Inventory of Executive Function (BRIEF), ^2^ a standardized instrument designed to assess core aspects of executive control in everyday contexts. The measure captures multiple domains, including impulse control, shifting between tasks or mental sets, holding and manipulating information in working memory, planning and organizing, managing emotional responses, initiating tasks, maintaining orderly materials, and self-monitoring. A composite executive functioning score was calculated using the overall scale, which demonstrated excellent internal consistency across all assessed time points (α = .98).

**Imaginary Companion.** When children were 4 and 6 years old, parents were interviewed concerning whether their child had an imaginary friend. Responses were coded dichotomously (No = 0, Yes = 1). To ensure the validity of this classification, follow-up questions were used to confirm that the described companion did not stem from a misinterpretation or confusion with fictional characters or real individuals.

**Self-esteem***.* Children’s overall sense of self-worth was measured at multiple time points using developmentally appropriate instruments. At ages 6, 8, and 10, participants completed the Self-Description Questionnaire I (SDQ-I),^3^ which consists of eight items evaluating global self-esteem. Responses were recorded on a 5-point scale ranging from 1 (“not true of me”) to 5 (“very true of me”), (α=.82-.87). For older children (ages 12 and 14), we used the Global Self-Worth subscale from the Self-Perception Profile for Adolescents—Revised (SPPA-R),^4,5^ which includes five items assessing general self-esteem. Items were rated on a 4-point Likert scale from 1 (“describes me very poorly”) to 4 (“describes me very well”), yielding internal consistency estimates ranging from .79 to .84.

**Personality Traits**. Between the ages of 10 and 14, children completed the Norwegian adaptation of the Big Five Inventory (BFI).^6^ a self-report measure designed to assess five core personality dimensions, Conscientiousness (α=.65-.77), Agreeableness (α=.64-.72), Neuroticism (α=.59-.81), Extraversion (α=.54-.75), and Openness (α=.70-.74).

**Emotion Regulation.** The teacher who knew the child best completed the Emotion Regulation Checklist (ERC)^7^ at ages 6-14. The instrument includes two subscales: one reflecting general emotion regulation abilities and the other assessing emotional lability and negativity. Given that the latter overlaps conceptually with personality traits already measured through the Big Five Inventory, only the Emotion Regulation subscale was included in analyses (8 items; α=.64-.74). It captures aspects such as positive emotionality (e.g., “Is a cheerful child”), interpersonal emotional interest (e.g., “is empathic towards others”), absence of negative emotions (e.g., ‘seems sad or listless’) and restricted emotional expression (e.g., ‘displays flat affect’).

**Social Skills**. Teachers rated children’s social functioning using instruments tailored to different age ranges. From ages 4 to 10, the 30-item Social Skills Rating System (SSRS)^8^ was administered, providing scores across key domains such as assertion, cooperation, and self-control (α =.89-.93). For assessments at ages 12 to 14, the updated Social Skills Improvement System (SSIS),^9^ was used. This version expands on the original SSRS by incorporating additional components like communication skills, empathy, and social engagement (α = .96).

**Social Withdrawal.** Social reticence in peer contexts was assessed through teacher reports using the Conflicted Shyness subscale of the Child Social Preference Scale,^10^ administered at ages 6 through 14. This subscale captures ambivalent social behavior, characterized by the desire to engage with peers coupled with hesitancy or avoidance—often referred to as approach-avoidance conflict. An example item includes: “The child circles other children’s play without participating” (α=.82-.89).

**Attachment. Ages 4 and 6: The Manchester Child Attachment Story Task (MCAST).** At ages 4 and 6, attachment patterns were assessed using the Manchester Child Attachment Story Task (MCAST),^11^ which is appropriate for children aged 4 to 8. The MCAST is a doll-play task in which one doll represents the child and another represents the parent. The child is presented with four narrative vignettes that begin with neutral, everyday scenarios but escalate to distressing situations, such as waking up alone after a nightmare. After each scenario reaches a critical point, the child is prompted with the question, “What happens next?” to encourage them to complete the story. The child is subsequently asked about the emotions of the child and the parent doll.

The child’s narrative and behavior in the vignettes are coded according to primary attachment strategies (A, B, C, or D), with secondary strategies assigned where appropriate. A mean attachment score was calculated across the four vignettes.^12^ Here, we used the secure attachment and disorganized attachment scores. All MCAST sessions were videotaped for later recoding. To assess reliability, a random subset of 157 videos was recoded by independent, blinded raters, yielding strong inter-coder reliability for secure attachment (ICC = .84) and disorganized attachment (ICC = .74).

***Ages 10-14: Middle Childhood Attachment Strategies Coding System (MCAS).*** At ages 10, 12, and 14, attachment strategies were assessed using the Middle Childhood Attachment Strategies Coding System (MCAS).^13^ In this task, each child and one parent were videotaped while engaging in a structured interaction designed to elicit conflict resolution. The dyads were instructed to identify and discuss the most significant area of disagreement for 8 minutes. While they were free to choose the topic, a list of common areas of conflict, such as chores, bedtimes, screen time, homework, and clothing, was provided as a guide. Based on the secure base concept, the MCAS categorizes attachment behaviors into four patterns identified during infancy: secure, avoidant, ambivalent, and disorganized-disoriented. A 9-point rating scale was used to evaluate the degree to which each behavior reflected the attachment strategy, with ratings ranging from 1 = *one or slight/isolated minor evidence of a specific pattern that does not characterize the child’s behavior and interaction overall* to 9=*marked and persistent evidence of a specific pattern that predominantly characterizes the child’s behavior and interaction*. A random selection of 315 videos from ages 10 to 14 were recoded by independent, blinded raters to assess reliability, with inter-coder reliability coefficients of ICC = .71 for secure attachment and ICC = .66 for disorganized attachment.

**Symptoms of Psychiatric Disorders.** At ages 4 to 6, parents were interviewed using the Preschool Age Psychiatric Assessment (PAPA),^14^ while at ages 8 to 14, both parents and children participated in separate interviews using the Child and Adolescent Psychiatric Assessment (CAPA).^15^ These semi-structured interviews include both mandatory questions and optional follow-up probes, with interviewers continuing to inquire until a definitive judgment can be made regarding the presence of a symptom. A primary reference period of 3 months was used, during which symptom onset, duration, and intensity were recorded. Symptoms were considered present if either the child or parent reported them. To create composite measures, the number of symptoms for various emotional disorders, including major depressive disorder, dysthymia, separation anxiety, social anxiety, generalized anxiety disorder, and specific phobias, were summed to form an index of emotional disorder symptoms. Similarly, the total number of symptoms for oppositional defiant disorder and conduct disorder were summed to create an index for behavioral disorders. A random subset of interviews (88 PAPA and 279 CAPA) were recoded by blinded raters, resulting in strong interrater reliability, with ICC values of .86 and .82 for emotional disorder symptoms and .90 and .83 for behavioral disorder symptoms. Additionally, the PAPA (but not the CAPA) includes 28 items assessing the B-criteria and 4 items for the A-criteria of autism spectrum disorder. These items were combined to form a composite score at ages 4 and 6, which demonstrated good reliability (ICC = .83).

**Serious Negative Life Events.**  Both parents (at all ages) and children (from age 8) were asked to report any experience of 26 specific life events over the previous two years. These events included traumatic occurrences such as the death of a close adult, a serious fall, exposure to violence or death, and experiences of physical and/or sexual abuse. An event was considered to have occurred if it was reported by either the child or the parent.

**Bullying Victimization.** The child’s primary teacher completed the 5-item Olweus Bully Victim Questionnaire (OBVQ)^16^ at 6 to 14 to evaluate both direct and indirect bullying experiences over the past 3 months. Items were rated on a 5-point Likert scale, with responses ranging from 1 (“never”) to 5 (“every day”). Midpoint values were used to calculate an estimate of the frequency of bullying incidents per month.

**Parental Personality Disorder Traits.** Parental personality disorder traits were assessed using the DSM-IV and ICD-10 Personality Questionnaire,^17^ a self-report measure completed by parents when their children were 4, 6, and 14 years old. Composite scores were calculated for each of the three personality disorder clusters (A, B, and C), with internal consistency estimates ranging from θ = .88 to .95.

**Supplementary Table 1.** *Sample Characteristics (%) of Participants at the Time of Study Enrollment (T1).*

| Characteristic |  | % |
| --- | --- | --- |
| Sex of child | Male | 49.1 |
|  | Female | 50.9 |
| Sex of parent informant | Male | 15.2 |
|  | Female | 84.8 |
| Ethnic origin of biological mother | Norwegian | 93.0 |
|  | Western countries | 2.7 |
|  | Other countries | 4.3 |
| Ethnic origin of biological father | Norwegian | 91.0 |
|  | Western countries | 5.8 |
|  | Other countries | 3.2 |
| Biological parents’ marital status | Married | 56.3 |
|  | Cohabitating >6 months | 32.6 |
|  | Separated | 1.7 |
|  | Divorced | 6.8 |
|  | Widowed | 0.2 |
|  | Cohabitating <6 months | 1.1 |
|  | Never lived together | 1.3 |
| Informant parent’s occupational level | Leader | 5.7 |
|  | Professional, higher level | 25.7 |
|  | Professional, lower level | 39.0 |
|  | Formally skilled worker | 26.0 |
|  | Farmer/fisherman | 0.5 |
|  | Unskilled worker | 3.1 |
| Parent’s highest completed education | Did not complete junior high school | 0.0 |
|  | Junior high school (10^th^ grade) | 0.6 |
|  | Some educational after junior high school | 6.1 |
|  | Senior high school (13^th^ grade) | 17.3 |
|  | Some education after senior high school | 3.4 |
|  | Some college or university education | 7.6 |
|  | Bachelor’s degree | 6.2 |
|  | College degree (3-4 years study) | 33.6 |
|  | Master’s degree or similar | 20.3 |
|  | PhD completed or ongoing | 4.4 |
| Households’ gross annual income | 0-225´ NOK (0-21´ USD) | 3.3 |
|  | 225´-525´ NOK (21´-49´ USD) | 18.4 |
|  | 525´-900´ NOK (49´-85´ USD) | 51.6 |
|  | 900´+ NOK (85´+ USD) | 26.7 |

**Supplementary Table 2**

*Estimated Prevalence of Cluster A Traits in the Population Below and Above the Diagnostic Threshold (n=647)*

| Traits | Below  threshold | Above threshold |
| --- | --- | --- |
| **Paranoid Personality Disorder** |  |  |
| Suspects, without sufficient basis, that others are exploiting, harming, or deceiving him or her. | 3.6 | 1.1 |
| Is preoccupied with unjustified doubts about the loyalty or trustworthiness of friends or associates. | 7.0 | 1.7 |
| Is reluctant to confide in others because of unwarranted fear that the information will be used maliciously against him or her. | 5.7 | 1.1 |
| Reads hidden demeaning or threatening meanings into benign remarks or events. | 1.9 | 0.2 |
| Persistently bears grudges (i.e., is unforgiving of insults, injuries, or slights). | 5.4 | 0.9 |
| Perceives attacks on his or her character or reputation that are not apparent to others and is quick to react angrily or to counterattack. | 2.9 | 0.8 |
| Has recurrent suspicions, without justification, regarding fidelity of spouse or sexual partner. | 2.5 | 0.5 |
| **Schizoid Personality Disorder** |  |  |
| General disinterest in social and personal relationships | 0.6 | 0.0 |
| Preference for solitude; almost always chooses solitary activities | 2.8 | 2.2 |
| Little to no interest in sexual experiences with another person | 1.7 | 0.6 |
| Inability to take pleasure in most activities | 1.7 | 0.6 |
| Emotional Coldness | 3.3 | 0.8 |
| Indifference to praise or criticism | 2.0 | 0.8 |
| **Schizotypal Personality Disorder** |  |  |
| Ideas of reference (notions that everyday occurrences have special meaning or significance personally intended for or directed to themselves) | 9.6 | 1.6 |
| Odd beliefs or magical thinking (e.g., believing in clairvoyance, telepathy, or a sixth sense; being preoccupied with paranormal phenomena) | 5.4 | 0.8 |
| Unusual perceptual experiences (e.g., hearing a voice whispering their name) | 7.9 | 2.0 |
| Odd thought and speech (e.g., that is vague, metaphorical, excessively elaborate, or stereotyped) | 5.4 | 1.7 |
| Suspicions or paranoid thoughts | 10.4 | 2.3 |
| Lack of close friends or confidants, except for 1st-degree relatives | 3.6 | 0.8 |
| Excessive social anxiety that does not lessen with familiarity and is related mainly to paranoid fears | 4.9 | 1.3 |

Note: Traits noted through behavior that could not easily be assessed during an interview were not assessed.

**Supplementary Table 3.** *Descriptive of the Growth Parameters of Predictors of Cluster A Traits*

|  |  | Growth parameters | | | Model fit | | | | |
| --- | --- | --- | --- | --- | --- | --- | --- | --- | --- |
| Predictor (age range in years) | *n* | Inter-cept | Slope | *p*-value Slope | χ^2^ | *df* | *p-*value | CFI | RMSEA  (90% CI) |
| Child factors | | | | | | | | | |
| Oddity^a^(4-14) | 956 | 1.04 | 0.00 | .367 | 12.20 | 10 | .272 | .980 | .020(.000, .038) |
| Executive functioning problems^a^ (6-14) | 851 | 88.28 | -0.11 | .33 | 38.54 | 10 | .006 | .948 | .059(.041, .079) |
| Imaginary friend^a^ (4-6) | 1004 | 0.20 | -0.01 | .475 |  |  |  | N.A. |  |
| Self-esteem^b^ (6-10) | 805 | 3.5 | 0.24 | <.001 | 0.00 | 0 | N.A. | N.A. | . |
| Self-esteem^a^ (12-14) | 681 | 3.56 | -0.16 | <.001 | 0.00 | 0 | N.A. | N.A. |  |
| Openness^a^ (10-14) | 719 | 3.78 | -0.07 | <.001 | 1.10 | 1 | .295 | 1.000 | .012(.000, .100) |
| Conscientiousness^a^ (10-14) | 719 | 3.67 | -0.02 | .003 | 10.78 | 1 | .001 | .971 | .117(.061, .184) |
| Agreeableness^a^ (10-14) | 719 | 4.18 | -0.04 | <.001 | 2.29 | 1 | .130 | .994 | .042(.000, .118) |
| Extroversion^a^ (10-14) | 719 | 3.60 | 0.01 | .249 | 4.76 | 1 | .029 | .988 | .072(.019, .143) |
| Neuroticism^a^ (10-14) | 719 | 2.51 | -0.02 | .041 | 10.24 | 1 | .001 | .965 | .113(.058, .181) |
| Emotion regulation^b^ (6-14) | 850 | 3.37 | -0.03 | <.001 | 34.44 | 7 | <.001 | .912 | .068(.046, .091) |
| Social-relational and environmental factors | | | | | | | | | |
| Social competence^b^ (4-10) | 997 | 2.96 | 0.01 | <.001 | 5.83 | 3 | .120 | .993 | .031(.000, .068) |
| Social competence^b^ (12-14) | 670 | 3.20 | -0.05 | <.001 | 0.00 | 0 | N.A. | N.A. |  |
| Social withdrawal^b^ (6-14) | 851 | 44.80 | -0.01 | .310 | 24.43 | 7 | .001 | .932 | .054(.032, .078) |
| Secure attachment^b^ (4-6) | 919 | 1.57 | 0.43 | <.001 | 0.00 | 0 | N.A. | N.A. |  |
| Secure attachment^b^ (10-14) | 679 | 1.92 | .001 | .995 | .061 | 1 | .435 | 1.000 | .000(.000, .093) |
| Disorganized attachment^a^ (4-6) | 919 | 0.19 | -.06 | <.001 | 0.00 | 0 | N.A. | N.A. |  |
| Disorganized attachment^a^ (10-14) | 717 | 1.58 | -.10 | <.001 | 0.12 | 1 | .730 | 1.000 | .000(-000, .070) |
| Negative life events^b^ (4-14) | 1068 | .12 | 0.03 | <.001 | 16.02 | 10 | .099 | .957 | .024(.000, .045) |
| Victimization from bullying, self-reported^b^ (6-14) | 850 | 4.57 | -0.24 | .001 | 8.29 | 7 | .310 | .941 | .015(.000, .046) |
| Parental cluster A symptoms^a^ (4,6,14) | 953 | .92 | -0.01 | .226 | .61 | 1 | .436 | 1.000 | .000(.000, .078) |
| Parentalcluster B symptoms^a^ (4,6,14) | 953 | 1.25 | -0.01 | <.001 | .65 | 1 | .421 | 1.000 | .000(.000, .079) |
| Parental cluster C symptoms^a^ (4,6,14) | 953 | 1.48 | -0.01 | <.001 | .02 | 1 | .896 | 1.000 | .000(.000, .039) |
| Psychopathology | | | | | | | | | |
| Emotional disorder symptoms^a^ (6-14) | 1040 | 6.16 | 0.38 | <.001 | 22.00 | 16 | .143 | .988 | .019(.000to.037) |
| Behavioral disorder symptoms^a^ (6-14) | 1040 | 5.45 | 0.00 | .932 | 41.94 | 15 | <.001 | .946 | .042(.027to.057) |
| Autism spectrum symptoms^a^ (4-6) | 1036 | 1.07 | -0.01 | .834 | N.A. | | | | |

*Note:* ^a^Linear growth model, ^b^Latent basis growth models. CFI=Comparative Fit Index, RMSEA=Root Mean Square Error of Approximation, CI=confidence interval, N.A.=Not applicable.

**References**

1. De Clercq B, Verbeke L, De Caluwé E, Vercruysse T, Hofmans J. Understanding adolescent personality pathology from growth trajectories of childhood oddity. *Dev Psychopathol*. Oct 2017;29(4):1403-1411. doi:10.1017/s0954579417000347

2. Gioia GA, Isquith PK, Guy SC, Kenworthy L. Behavior Rating Inventory of Executive Function. *Child Neuropsychology*. 2000;6(3):235-238. doi:10.1076/chin.6.3.235.3152

3. Marsh HW, Barnes J, Cairns L, Tidman M. Self-description questionnaire: Age and sex effects in the structure and level of self-concept for preadolescent children. *Journal of Educational Psychology*. 1984;76(5):940–956. doi:10.1037/0022-0663.76.5.940

4. Harter S. *Manual for the self-perception profile for adolescents*. University of Denver; 1988.

5. Wichstrøm L. Harters Self-Perception Profile for Adolescents - Reliability, Validity, and Evaluation of the Question Format. *J Pers Assess*. AUG 1995;65(1):100-116.

6. Soto CJ, John OP, Gosling SD, Potter J. The developmental psychometrics of big five self-reports: Acquiescence, factor structure, coherence, and differentiation from ages 10 to 20. *J Pers Soc Psychol*. Apr 2008;94(4):718-737. doi:10.1037/0022-3514.94.4.718

7. Shields A, Cicchetti D. Emotion regulation among school-age children: The development and validation of a new criterion Q-sort scale. *Developmental Psychology*. Nov 1997;33(6):906-916.

8. Gresham FM, Elliot SN. *Social Skills Rating System*. American Guidance Service; 1990.

9. Gresham FM, Elliott SN, Vance MJ, Cook CR. Comparability of the Social Skills Rating System to the Social Skills Improvement System: Content and Psychometric Comparisons Across Elementary and Secondary Age Levels. *Sch Psychol Q*. Mar 2011;26(1):27-44. doi:10.1037/a0022662

10. Coplan RJ, Prakash K, O'Neil K, Armer M. Do you "want" to play? Distinguishing between conflicted shyness and social disinterest in early childhood. *Developmental Psychology*. Mar 2004;40(2):244-258. doi:10.1037/0012-1649.40.2.244

11. Green JM, Stanley C, Smith V, Goldwyn R. A new method for evaluating attachment representations in young school-age children: The Manchester Child Attachment Story Task. *Attachment & Human Development*. 2000;2:48-70.

12. Viddal KR, Berg-Nielsen TS, Belsky J, Wichstrom L. Change in Attachment Predicts Change in Emotion Regulation Particularly Among 5-HTTLPR Short-Allele Homozygotes. Article. *Developmental Psychology*. Jul 2017;53(7):1316-1329. doi:10.1037/dev0000321

13. Brumariu LE, Giuseppone KR, Kerns KA, et al. Middle Childhood Attachment Strategies: validation of an observational measure. Article. *Attachment & Human Development*. 2018;20(5):491-513. doi:10.1080/14616734.2018.1433696

14. Egger HL, Erkanli A, Keeler G, Potts E, Walter BK, Angold A. Test-retest reliability of the Preschool Age Psychiatric Assessment (PAPA). *J Am Acad Child Adolesc Psychiatry*. May 2006;45(5):538-549.

15. Angold A, Costello EJ. The Child and Adolescent Psychiatric Assessment (CAPA). *J Am Acad Child Adolesc Psychiatry*. JAN 2000;39(1):39-48.

16. Solberg ME, Olweus D. Prevalence estimation of school bullying with the olweus bully/victim questionnaire. *Aggressive Behavior*. 2003/04/22 2003;29(3):239–268. doi:10.1002/ab.10047

17. Ottosson H, Bodlund O, Ekselius L, et al. DSM-IV and ICD-10 personality disorders: a comparison of a self-report questionnaire (DIP-Q) with a structured interview. Article. *Eur Psychiatry*. 1998;13(5):246-253. doi:10.1016/s0924-9338(98)80013-8
